# Tracking neighbours promotes the coexistence of large carnivores

**DOI:** 10.1038/srep23198

**Published:** 2016-03-16

**Authors:** José Vicente López-Bao, Jenny Mattisson, Jens Persson, Malin Aronsson, Henrik Andrén

**Affiliations:** 1 Research Unit of Biodiversity (UO/ CSIC/PA), Oviedo University, 33600 Mieres, Spain; 2 Grimsö Wildlife Research Station, Department of Ecology, Swedish University of Agricultural Sciences, 73091 Riddarhyttan, Sweden; 3 Norwegian Institute of Nature Research, 7485 Trondheim, Norway

## Abstract

The study of competition and coexistence among similar interacting species has long been considered a cornerstone in evolutionary and community ecology. However, understanding coexistence remains a challenge. Using two similar and sympatric competing large carnivores, Eurasian lynx and wolverines, we tested the hypotheses that tracking among heterospecifics and reactive responses to potential risk decreases the probability of an agonistic encounter when predators access shared food resources, thus facilitating coexistence. Lynx and wolverines actively avoided each other, with the degree of avoidance being greater for simultaneous than time-delayed predator locations. Wolverines reacted to the presence of lynx at relatively short distances (mean: 383 m). In general, lynx stayed longer, and were more stationary, around reindeer carcasses than wolverines. However, when both predators were present at the same time around a carcass, lynx shortened their visits, while wolverine behavior did not change. Our results support the idea that risk avoidance is a reactive, rather than a predictive, process. Since wolverines have adapted to coexist with lynx, exploiting lynx-killed reindeer carcasses while avoiding potential encounters, the combined presence of both predators may reduce wolverine kill rate and thus the total impact of these predators on semi-domestic reindeer in Scandinavia. Consequently, population management directed at lynx may affect wolverine populations and human-wolverine conflicts.

The study of competition and coexistence among similar species has long been considered a cornerstone in evolutionary and community ecology^1^,^2^,^3^,^4^. Inter-specific competition, either exploitation or interference competition, is a major force affecting species and shaping community structure^2^,^3^,^5^,^6^,^7^. During the last three decades, mammalian carnivores have emerged as a paradigmatic group of model species to understand intraguild competition (kleptoparasitism, intraguild predation, avoidance behaviour)^8^, as well as the behavioural mechanisms facilitating coexistence, mainly focused on niche theory^9^,^10^,^11^, and dietary niche segregation in particular^12^,^13^,^14^. Such interest has probably been reinforced by intraguild predation processes^7^ as well as top-down ecosystem effects triggered by large carnivores^15^,^16^.

After several decades of documenting competition, and its consequences, understanding how similar interacting species (e.g. similar body size and/or diet) manage to coexist remains challenging. Traditionally, temporal partitioning of activity, spatial (i.e. distance-sensitive avoidance), and habitat segregation have been proposed as the main mechanisms to reduce competition or the probability of risky encounters between competing carnivores^17^,^18^,^19^. However, competing carnivores with different degrees of overlapping diets often overlap in time, space, and habitat^20^,^21^,^22^,^23^,^24^. This suggests that the competitive dynamics and underlying behavioural mechanisms facilitating coexistence are more complex than previously shown^22^,^25^,^26^,^27^,^28^.

Focusing the study of animal interactions only on static processes, such as habitat or temporal partitioning, might overlook subtler behavioural tactics also facilitating coexistence. The integration of fine-scale movement patterns therefore becomes critical to identify mechanisms promoting coexistence^29^. Active avoidance^23^,^27^,^30^, which is independent of fixed temporal and spatial windows and habitat characteristics, is gaining growing attention as an important mechanism reducing risk of encounters and agonistic interactions among carnivores. For example, new research on the response of carnivores to the nearest recent location of their competitors at a fine scale suggests that interacting species have real-time spatial awareness of the occurrence of competitors, and adapt their behaviour accordingly^23^. Active avoidance strategies may therefore be pivotal in allowing landscape-sharing while minimizing the potential risk of coexistence without reducing access to resources^23^,^25^,^28^. Fine-scale active avoidance (i.e. reactive response to a potentially risky encounter based on animals’ knowledge of real-time risks)^25^, thus emerges as an important candidate mechanism facilitating carnivore coexistence. However, only a few examples and critical tests are available for understanding how this mechanism may operate and the underlying behavioural adaptations and consequences for interacting species^22^,^23^,^25^.

Access to food resources (or to limited resources, such as water ponds in arid landscapes) may be the predominant form of interference competition^31^ and among large carnivores this will often take place at prey carcasses^32^,^33^. Here, we examined fine spatio-temporal patterns of active avoidance and shifting behaviours as important drivers of coexistence between two similar competing large carnivores, the Eurasian lynx (*Lynx lynx*) and the wolverine (*Gulo gulo*) (Fig. 1), when sharing the same food resource. The two predators are of similar size (in the study area: Eurasian lynx = 17–22 kg, wolverine = 10–14 kg) and share the same boreal landscape^34^. Lynx and wolverines show no evidence of temporal or spatial segregation^21^, select similar habitats^24^, and have highly overlapping diets^33^. We took advantage of the fact that the main source of food for both predators in the study area was semi-domestic reindeer (*Rangifer tarandus*). Furthermore, while lynx is an obligate predator, the wolverine is a facultative scavenger and predator who frequently scavenges on reindeer carcasses killed by lynx^33^. Therefore, interference competition around reindeer carcasses killed by lynx is expected to occur as lynx and wolverine return to reindeer carcasses multiple times to feed over several days following a predation event^35^.

We tested the hypotheses that tracking among heterospecifics and reactive responses to potential risk decreases the probability of an agonistic encounter when predators access shared food resources, thus facilitating coexistence. We predicted that i) lynx and wolverines will react to the actual risk of encountering heterospecifics^23^. Thus, the probability of a predator visiting a shared reindeer carcass will be influenced by the actual location of heterospecifics regarding the carcass as well as the distance between the competitors. Furthermore, according to a reactive response hypothesis^25^, we predicted that ii) the strength of such behavioural responses will be stronger for simultaneous (high probability of an encounter; immediate risk) compared to time-delayed predator locations; which would reflect a predictive response to a potential for a risky encounter (*sensu* landscape of risk)^25^. We also predicted that iii) lynx and wolverines will adapt their behaviour when visiting lynx-killed reindeer carcasses according to the presence/absence of heterospecifics nearby^36^.

## Results

Lynx and wolverines were monitored on average 4 days after a kill event occurred (range 2–8; a total of 61 sampling days). A mean of 175 lynx and wolverine pairs of simultaneous 30-min locations were used per reindeer carcass (range 83–336). During this period, we detected 122 carnivore visits to reindeer carcasses killed by lynx; 46 visits of lynx, 51 visits of wolverines and 25 visits in which both predators were present in a 650 m buffer zone at the same time. Thirty-six percent of lynx locations were less than 1,000 m from the reindeer carcasses (Fig. S3). Interestingly, even though lynx killed the reindeer, as much as 29% of the wolverine locations were less than 1,000 m from the carcasses as well (Fig. S4). However, lynx and wolverines did not meet frequently. Only 9.7% of simultaneous lynx and wolverine locations were located within 1,000 m of each other, with 4.4% less than 500 m apart (Fig. S5).

**Figure 2:**
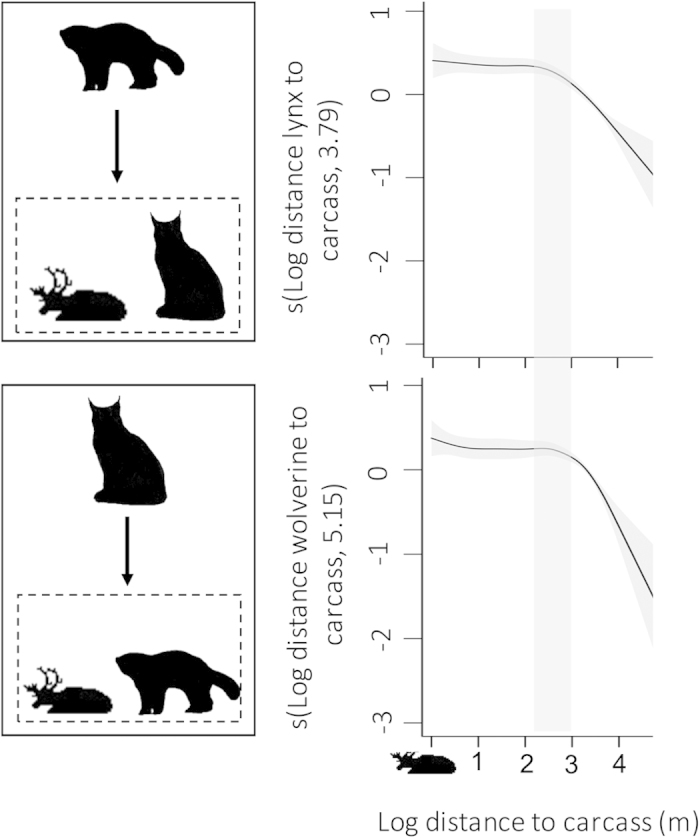
Effects of the location of heterospecifics in relation to reindeer carcasses (boxes with dotted lines) on the distance between wolverine (top) and lynx (bottom) to reindeer carcasses. Shaded areas represent 95% confidence intervals. The influence of heterospecifics was stronger below ca. 1,000 m (vertical grey bar).

The probability that a wolverine was close to a reindeer carcass was significantly influenced by the location of the lynx in relation to the carcass (Table 1, Fig. 2; Fig. S1). The same behavioural response was found when lynx was the focal species (Table 1, Fig. 2). The influence of heterospecifics increased for both predators when they were less than 1,000 m from the carcasses (Fig. 2). Accordingly, the distance between the two predators significantly and negatively influenced the distance between the focal predator and the reindeer carcass (Table 1). The strength of the influence of heterospecifics was higher for simultaneous (real-time) locations than for 30 min time-delayed locations (based on BIC; Table 1).

**Figure 3:**
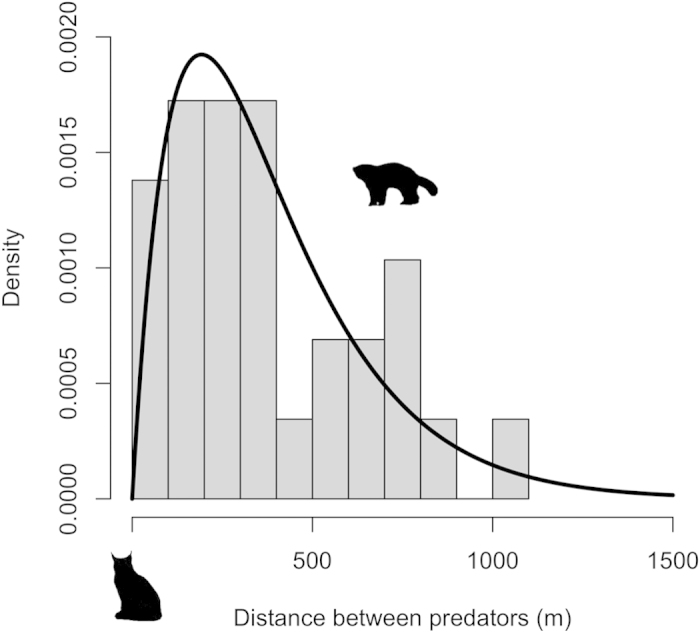
Wolverine reaction distances to lynx presence. Grey bars represent the observed frequency distributions of reaction distances (n = 29) and the line best-fitting the probability density curve following a Gamma distribution (see Table S2).

Potential visual contacts between lynx and wolverines were very rare (only 21 cases, or 1% of pairs of total simultaneous locations; n = 2,133). After a visual contact (in 96% of cases lynx was closest to the carcass), wolverines most often moved away (80% of cases), while lynx or both predators moved away from carcasses in 10% of cases, respectively. We found 213 pairs of simultaneous locations where lynx and wolverines were within 1,500 m of the reindeer carcasses at the same time (10% of pairs of total simultaneous locations; n = 2,133). Focusing on the most sensitive species after a visual contact (wolverine), we detected a real-time reactive behavioural response of wolverines to avoid lynx (Fig. 3). The range of distances where wolverines reacted to the presence of lynx (n = 29, note that here we are considering more events than just visual contacts) ranged from 45 to 1,059 m (Fig. 3). Of these distances, only 13% occurred at less than 100 m (Fig. 3). The kernel estimate confirms that wolverines reacted to the presence of lynx at relatively short distances (Fig. 3), with a mean reaction distance of 383 m. The shape of the kernel generated by wolverine reaction distances was described by a Gamma distribution (based on AIC, Table S2) and was characterized by a peak between 100 and 400 m (Fig. 3).

**Figure 4:**
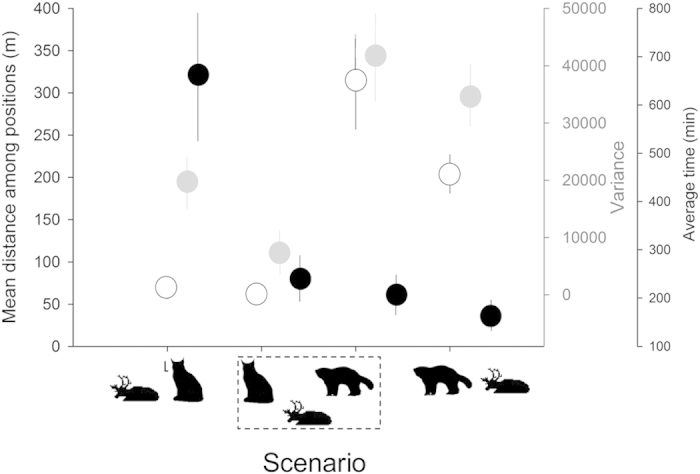
Lynx and wolverine behaviour, represented by the average time of a visit (black dots with s.e. bars), the mean distance among consecutive locations (open dots with s.e. bars) and its variance (grey dots with s.e. bars), when they visited the reindeer carcasses alone or they occurred simultaneously in the 650 m buffer zone.

The behaviour of lynx around reindeer carcasses was different compared to wolverines (Mann-Whitney U-tests, all *P* for mean distance among locations, its variance, and the average time per visit <0.01; Fig. 4). For lynx, mean ( ± s.e.) distance among all locations within a visit was 84 ( ± 10) m, and the mean ( ± s.e.) duration of a visit was 662 ( ± 158) min. For wolverines, these numbers were 204 ( ± 22) m and 167 ( ± 27) min, respectively (Fig. 4). When both predators were around the reindeer carcass at the same time, lynx decreased their movements (lower variance) and shortened their visits (the average duration of lynx visits was reduced by 3-fold). However, only the observed decrease in the duration of visits was marginally significant (Mann-Whitney U-tests, *P* = 0.07; rest of comparisons *P* > 0.33; Fig. 4). Wolverines, on the other hand, did not significantly change the duration of the visits or the mean distance among locations and its variance (Mann-Whitney U-tests all *P* > 0.30; Fig. 4).

## Discussion

In this study, using dynamic approaches at fine spatio-temporal scales, we shed light onto subtle mechanisms facilitating large carnivore (lynx and wolverine) coexistence. We took advantage of the very particular scenario of the simultaneous use of the same food resources by competing predators (lynx-killed reindeer carcasses). This allowed us to critically test for the existence of interference competition as well as the behavioural mechanisms facilitating synchronous resource use.

Lynx and wolverines share the same landscape and habitat characteristics, overlap their diet niche, and even use the same prey carcasses^21^,^24^,^33^. However, only weak evidence of partial exploitation competition has been found^33^. A priori, this previous information together with our results, showing that wolverines were located closer than 1,000 m from the lynx-killed reindeer carcasses with a similar frequency as lynx (Figs S3 and S4) could erroneously suggest the lack of interference between the two predators^33^. However, interference between these predators emerged when we considered fine spatio-temporal scales and used intensive monitoring approaches centred on the use of shared resources. Lynx primarily used reindeer carcasses in the first few days after a kill event, while wolverines used these carcasses long after lynx abandoned the carcasses^33^ (Fig. S2). Focusing on events when lynx and wolverines used a reindeer carcass simultaneously, we detected an active avoidance of the other species (Fig. 2; Fig. S1). The distance between the predators significantly influenced the decision to visit a carcass for both predators, particularly within 1,000 m around the carcasses (Fig. 2). Although these patterns were constant regardless of the time frame considered, the strength of the influence of heterospecifics was, as predicted, greater when we considered simultaneous real-time locations.

Although intra-guild killing or temporal and spatial segregation have not been reported between these species^37^,^38^, potential harm associated with interactions may be an important factor driving active avoidance between these two solitary predators. Tracking among heterospecifics and reactive responses to potential risky encounters^23^,^25^ decreased the probability of an encounter, thus promoting fine-scale coexistence between lynx and wolverine. Both lynx and wolverines reacted to the nearest recent location of heterospecifics, indicating that both predators exhibit spatial awareness of the presence of heterospecifics at relatively short distances^23^. Accordingly, these results, together with the reactive response observed in wolverines at short distances from lynx, and the similar habitat preferences for both species^24^, supports the idea that risk avoidance is reactive, rather than a predictive process^25^,^28^. This is consistent with Broekhuis and colleagues (2013) who showed that cheetahs *(Acinonyx jubatus)* did not consistently show a predictive response to avoid habitats and areas with a high likelihood of encountering lions *(Panthera leo)* or spotted hyaenas *(Crocuta crocuta)* but instead, showed reactive responses adjusting their behaviour to the nearest position of competitors^25^. Reactive behavioural responses may also be an important behavioural mechanism explaining the persistence of large carnivores in some human-dominated landscapes^39^.

Wolverines approached lynx up to close distances before reacting and moving away. This suggests that wolverines may respond to immediate signals such as visual detection of lynx^27^, even after they have detected lynx by smell. This strategy may be advantageous for wolverines to track the presence of lynx. When both predators occurred at the same time in the buffer zone, lynx reduced their movements, probably in order to defend its prey; whereas wolverines kept moving around the reindeer carcasses, probably to track lynx presence more efficiently, disturb lynx or to obtain the carcass. In addition, encounter competition between the two predators affected the time lynx spent visiting the carcasses, suggesting that wolverine presence is a nuisance for lynx^33^. However, the average time of a wolverine visit did not change between scenarios, probably because wolverines continuously adopt a time-minimizing strategy^40^ when visiting prey carcasses from other predators such as lynx or wolves *(Canis lupus)*^41^. Additionally, constant short visits by wolverines could be explained by the fact that the caching behaviour of wolverines^42^ results in a continuous back and forth movement pattern between the carcass and cache sites.

Nevertheless, this scenario of coexistence may change if reindeer availability – influenced by reindeer husbandry decisions-change over time^43^. For lynx, in a system where reindeer are abundant, perhaps the cost/benefit ratio to kill a new reindeer is lower than a potentially risky encounter or fight with a wolverine. Lynx mainly eat soft parts, whereas wolverines can easily crush bones and eat skin and hooves. Lynx therefore provide increased resources for wolverines through increased scavenging opportunities^33^, leading to a facilitation process where wolverines may benefit from coexisting with lynx, which may have affected its fitness and population recovery in recent decades^44^,^45^. Similarly, wolf presence shifted the wolverine diet from reindeer to wolf-killed moose^41^. Although wolverines might not be dependent on other predators for survival or reproduction, the increased availability of scavenging opportunities and carrion in winter enhances the reproductive rate in wolverine populations^45^,^46^.

Understanding the mechanism and consequences of coexistence between large carnivores not only has substantial ecological relevance^16^, but also important management and conservation implications^47^. For example, since wolverines have adapted to coexist with lynx by exploiting lynx-killed reindeer while avoiding risky encounters, the combined presence of both predators may reduce wolverine kill rate and thus the total impact of lynx and wolverines on semi-domestic reindeers in Scandinavia^48^. Consequently, population management directed at lynx, such as lynx population control, may affect wolverine populations and human-wolverine conflicts.

## Material and Methods

### Study Area

Lynx and wolverines were studied in and around Sarek National Park, northern Sweden (Kvikkjokk: 67.80°N, 17.84°E). The study area is characterized by deep valleys starting at about 300 m a.s.l. and high mountainous plateaus with peaks up to 2,000 m a.s.l. The main vegetation at lower elevations consists of mixed conifer forest (Scots pine *Pinus sylvestris* and Norway spruce *Picea abies*) interspersed with numerous bogs and lakes, followed by hillsides and high elevation valleys of mountain birch forest (*Betula pubescens*), which form the tree line at 600–700 m a.s.l. The higher parts of the hillsides include low alpine tundra with dwarf birch (*Betula nana*) and willow shrubs (*Salix spp.*), succeeded by lower growing heaths, grass and meadows, to peaks and high plateaus of bare rock and glaciers. The ground is usually snow covered from November to May. The area includes important spring–autumn grazing pastures for semi-domestic reindeers, but groups of reindeer remain during the winter season^49^.

### Lynx, Wolverines and Reindeer Carcasses

We used simultaneous GPS-locations to investigate lynx and wolverine behaviour around reindeer carcasses being used simultaneously by both predators. We used an intensive schedule acquiring a location every 30 min between 15:00 in the afternoon and 09:00 in the morning CET (Central European Time). We used this sampling interval because this is the time-period when both species are most active^50^. GPS fix rate was 96% for lynx and 83% for wolverines in the study area, with a remarkably high proportion of 3D/3D + fixes, and fix rate was not influenced by forest cover^42^,^50^. Animals were captured on the ground or darted from a helicopter, immobilized with a mixture of ketamine and medetomidine following pre-established protocols, and equipped with downloadable GPS collars (Vectronic Aerospace GmbH, Berlin, Germany). Lynx and wolverines were captured and immobilized in accordance with strict handling protocols, examined and approved by the Swedish Animal Ethics Committee; and fulfilled the Swedish ethical requirements for research on wild animals.

We located reindeer carcasses killed by lynx by identifying clusters of lynx GPS locations using ArcGIS (ESRI, California, USA) during intensive fieldwork periods (6 weeks in March-April, July-August, and October-November in 2008, respectively, and 4 weeks in January and May in 2009, respectively^33^,^49^). The criterion used to define a potential cluster was two locations less than 100 m apart. Once a cluster was identified, we investigated it in the field to determine the occurrence of a kill event and the presence of a reindeer carcass. As lynx rarely scavenge, reindeer carcasses found at lynx clusters were classified as lynx-killed, if there were no signs indicating otherwise. We additionally used extra information, such as bite marks on the throat or tracks in the snow, indicative of a successful hunt to identify predation events correctly. The date and time of the predation event was set to the first GPS location of the lynx at the carcass.

During the study period, we did predation studies on 7 and 8 GPS-collared lynx and wolverines, respectively, during a total of 1417 days (all animals pooled). As a result, we found 206 reindeer carcasses of which 149 where lynx killed reindeer that were available for the wolverines to scavenge (in which they scavenged 68%)^33^. From this dataset, we focused the present study on those reindeer carcasses being used simultaneously by both predators (n = 15). Therefore, ca. 17,500 simultaneous GPS-locations from seven pairs of sympatric collared lynx (n = 3) and wolverines (n = 4), within an area of ca. 2,500 km^2^, were used to investigate lynx and wolverine behaviour around shared reindeer carcasses.

### Data Analyses

For all lynx-killed reindeer carcasses where we observed wolverine scavenging during lynx handling time^33^, we selected all lynx and wolverine simultaneous GPS locations in the following days after the kill event, until both the lynx and wolverines stopped visiting the carcasses, resulting in a subset of 2,133 pairs of simultaneous GPS locations for subsequent analyses. Focusing on lynx and wolverine dyads, starting from the estimated date and time of the kill event, we calculated the distances from UTM coordinates between consecutive lynx and wolverine locations and the location of the corresponding reindeer carcass, as well as the distance between simultaneous locations of the focal lynx and wolverine. Given the irregular topography of this mountain landscape, we calculated real distances using x, y and z coordinates.

Based on the distances between simultaneous lynx and wolverine locations, we identified potential visual contacts between both predators. To do this, we considered that two animals had a potential visual contact if they were less than 250 m apart in open sites, and less than 100 m apart in forested sites. Additionally, in forested sites, we used an extra cutting point of less than 50 m apart in summer to account for the effects of vegetation growth on visibility. However, in this mountainous and rugged landscape even when two animals are separated by less than 250 m, does not mean that a visual contact has occurred, due to the topography and vegetation. To refine this, we built three-dimensional maps considering a 50 × 50 m digital elevation model (Geographical Data Sweden, National Land Survey of Sweden) and overlapped it with high-resolution orthoimages of the study area. Thus, we were able to classify all short distance (<250 m) pairs of simultaneous lynx and wolverine locations as either a potential visual contact or not according to topography and vegetation. Every time we detected a potential visual contact in the dataset following such criteria, we recorded whether lynx or wolverines moved away from the location of the reindeer carcasses.

After a kill event, the median distance between lynx daybeds and reindeer carcasses is ca. 650 m^35^. Therefore, we used this distance to generate a buffer area around reindeer carcasses of 650 m radius (hereafter buffer zone) to study lynx and wolverine behaviour in the vicinity of the carcasses. We were interested in predator behaviour in two different scenarios: i) with only one of the predators present or ii) with both species present in the 650 m buffer zone at the same time. Every time an animal was located within the buffer zone, we categorized the event as a single (lynx or wolverine) or a multiple visit (both predators within the buffer zone at the same time). Next, for each visit to a carcass, we considered the total number of lynx/wolverine locations inside the buffer zone to calculate the minimum time (min) spent by each animal in the vicinity of the reindeer carcasses, estimated by multiplying the number of locations inside the buffer zone during the visit by 30 min. Those visits spanning the limits of the daily sampling period (from 15:00 to 9:00 CET) were not considered for subsequent analyses. For each individual visit, we calculated the mean distance among consecutive locations and its variance. Both parameters were used as proxies of the movement patterns of predators around the carcasses. For example, low values for the mean distance and the variance among consecutive locations would mean that an animal decreased its movements, whereas both high mean distances and variances would indicate that the animal was moving around the carcass while inside the buffer zone.

Moreover, to determine the existence of reactive real-time behavioural responses between lynx and wolverines regarding potential risky encounters while accessing a carcass, we selected all pairs of simultaneous lynx and wolverine locations within 1,500 m from the reindeer carcasses. We opted for at least double the radius used to generate the 650 m buffer zone because of the high olfactory potential in mammalian carnivores^51^. The animal’s decisions to avoid a potential agonistic encounter may happen at greater distances than for that needed for visual contact to occur. Considering all selected locations, we tracked the distance between the two predators simultaneously as well as their respective distance to the reindeer carcasses. When the distance between a lynx and a wolverine in time *t*_*+1*_ was longer than in *t*, we considered a reactive behavioural response to have occurred at *t*.

### Statistical Analyses

Since several lynx and wolverine dyads were studied repeatedly, we built Generalized Additive Mixed Models (GAMMs) with Gaussian error distributions and identity link to test for a non-linear influence of the spatial location of heterospecifics on the distance between a lynx or wolverine to a reindeer carcass. We also included the distance between the two predators as a covariate in the models. The magnitude of multicollinearity between the distance of the focal predator and the reindeer carcasses and the distance between predators was evaluated by considering the size of the variance inflation factor (VIF), which in all cases was less than 2.8. We log_10_-transformed all distances for analyses. The use of reindeer carcasses by lynx and wolverines varies over time^33^ (Fig. S2), and consecutive distances between predators and the reindeer carcasses, as well as distances between simultaneous and consecutive lynx and wolverine locations are temporally auto-correlated. For all lynx and wolverine locations, we calculated the time (h) elapsed between the estimated date and time of the kill event and the date and time when the animal location was taken (i.e. carcass exposure time). Thus, we ran the models accounting for non-independence in our repeated distance measurements for the same individuals using exposure time as a continuous autoregressive term *“corCAR1”* nested within each kill site event¸ and allowing the effect of exposure time to vary between kill events (i.e. time series)^52^,^53^. All distances were modeled using non-parametric smoothing splines. The appropriate degrees of freedom of the smoothers were selected automatically using cross-validation^54^. We fitted the same additive models separately for each species. Although the maximum distance recorded between a lynx or a wolverine and the reindeer carcass, was ca. 22 km and ca. 51 km, respectively, we ran all GAMMs considering a distance threshold around the reindeer carcasses of 10 km. We chose this distance because we expected that the influence of a competing predator affecting the decision process of its heterospecifics regarding whether to approach a carcass would relax in space. Thus, the final dataset for GAMMs contained 1,643 out of the 2,133 pairs of total simultaneous and consecutive lynx-wolverine locations. Furthermore, we ran a separate model with the same GAMM structure considering a time delay of 30 min between predator locations to test whether the influence of heterospecifics was stronger at real-time (i.e. simultaneous locations) compared to time-delayed locations. We used Bayesian Information Criterion (BIC) to identify the best candidate model (simultaneous vs. time-delayed model) explaining the influence of heterospecifics on predator approaches to carcasses^55^.

We used Mann-Whitney U-Tests to test for differences in the time spent and mean distance among locations, and its variance, for each predator species within the 650 m buffer zone,, according to whether lynx or wolverines visited the carcasses alone or if they occurred simultaneously within the buffer zone. The same statistical procedure was used to test for behavioural differences between predators visiting the carcasses. We used probability density functions (i.e. kernel estimates) to describe lynx-wolverine reactive behavioural response distribution around reindeer carcasses. We fitted the following density functions to the data on reactive distances: exponential, normal, lognormal, Student’s t, and gamma. We estimated the parameters of each distribution based on maximum-likelihood estimation, and Akaike Information Criterion (AIC) was used for model selection to identify the most appropriate distribution^56^. GAMMs were fitted using the ‘*mgcv*’ package^57^ within the R 3.1 statistical software^58^. The identity of the dyad and the identity of the reindeer carcasses were treated as random factors in all models. The probability density functions to describe the reactive behavioural responses were fitted using the package ‘*fitdistrplus’* in R^59^.

## Additional Information

**How to cite this article**: López-Bao, J. V. *et al.* Tracking neighbours promotes the coexistence of large carnivores. *Sci. Rep.* **6**, 23198; doi: 10.1038/srep23198 (2016).

## Supplementary Material

Supplementary Information

## Figures and Tables

**Figure 1:**
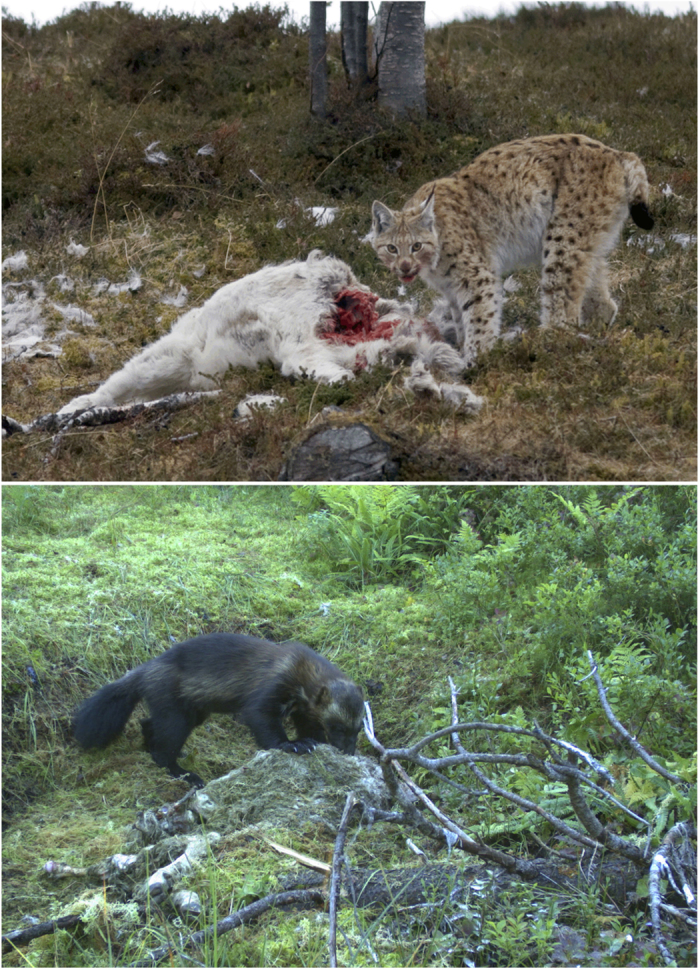
Eurasian lynx (*Lynx lynx*) and wolverine (*Gulo gulo*) feeding on prey. Pictures courtesy of Scandlynx@Ken Gøran Uglebakken (lynx) and viltkamera.nina.no (Norwegian Institute for Nature Research) (wolverine).

**Table 1:** Detailed results for generalized additive mixed models testing for the effect of the spatial location of heterospecifics on the distance between the carnivore and the reindeer carcass, based on 1,643 simultaneous locations for simultaneous (real-time) locations (A) and a time delay between predator locations of 30 min (B).

	WOLVERINE				LYNX			
	estimate	se	edf	*p*	estimate	se	edf	*p*
**A) Simultaneous (real-time) predator locations**								
*Parametric coefficients:*								
Intercept	3.07	0.05		<0.001	3.1	0.06		<0.001
*Smooth terms:*								
ƒ (Distance between predators)			5.67	<0.001			3.86	<0.001
ƒ (Distance competitor-carcass)			3.79	<0.001			5.15	<0.001
R^2^ (adjusted)	0.21				0.26			
BIC	**1721.9**				**1442.2**			
**B) 30-min time delay between predator locations**								
*Parametric coefficients:*								
Intercept	3.07	0.05		<0.001	3.02	0.07		<0.001
*Smooth terms:*								
ƒ (Distance between predators)			4.33	<0.001			2.12	<0.001
ƒ (Distance competitor-carcass)			3.89	<0.001			2.73	<0.001
R^2^ (adjusted)	0.14				0.16			
BIC	1865.2				1451.3			
*edf = estimated degrees of freedom.*								

For each species, models (A and B) were compared by using Bayesian Information Criterion (BIC). BIC tend to be more conservative and less prone to overparameterization than Akaike’s Information Criterion^55^. BIC of the best models are denoted in bold.
