# Supplementary material for: Tracking neighbours promotes the coexistence of large carnivores
Source: Sci Rep. 2016 Mar 16;6:23198. doi: 10.1038/srep23198 (PMC4793264; doi:10.1038/srep23198)
Supplement: Supplementary Information [file srep23198-s1.doc]

**Tracking neighbours promotes the coexistence of large carnivores**

José Vicente López-Bao, Jenny Mattisson, Jens Persson, Malin Aronsson & Henrik Andrén

**Figure S1.** Illustrative example showing the distance between consecutive lynx (black) and wolverine (blue) locations and a reindeer carcass in the first week after the lynx killed the reindeer.

**Figure S2.** Effect of exposure time on the location of lynx and wolverines in relation to reindeer carcasses.

**Figure S3.** Histogram showing the distribution of distances between lynx locations and the reindeer carcasses (n total days sampled = 61, all carcasses pooled). Grey bars denotes cases <1,000 m

**Figure S4.** Histogram showing the distribution of distances between wolverine locations and the reindeer carcasses (n total days sampled = 61, all carcasses pooled). Grey bars denotes cases <1,000 m.

**Figure S5.** Histogram showing the distribution of distances between simultaneous locations of lynx and wolverines (n total days sampled = 61, all carcasses pooled). Grey bars denotes cases <1,000 m

**Table S1.** Detailed results of generalized additive mixed models that test for non-linear relationships between wolverine and lynx positions regarding to the location of reindeer carcasses and exposure time (h).

**Table S2.** Best-fitting probability density functions to wolverine reaction distances to lynx encounters.

**Figure S1.** Illustrative example showing the distances between consecutive lynx (black) and wolverine (blue) locations and a reindeer carcass in the first week after a lynx kill event.


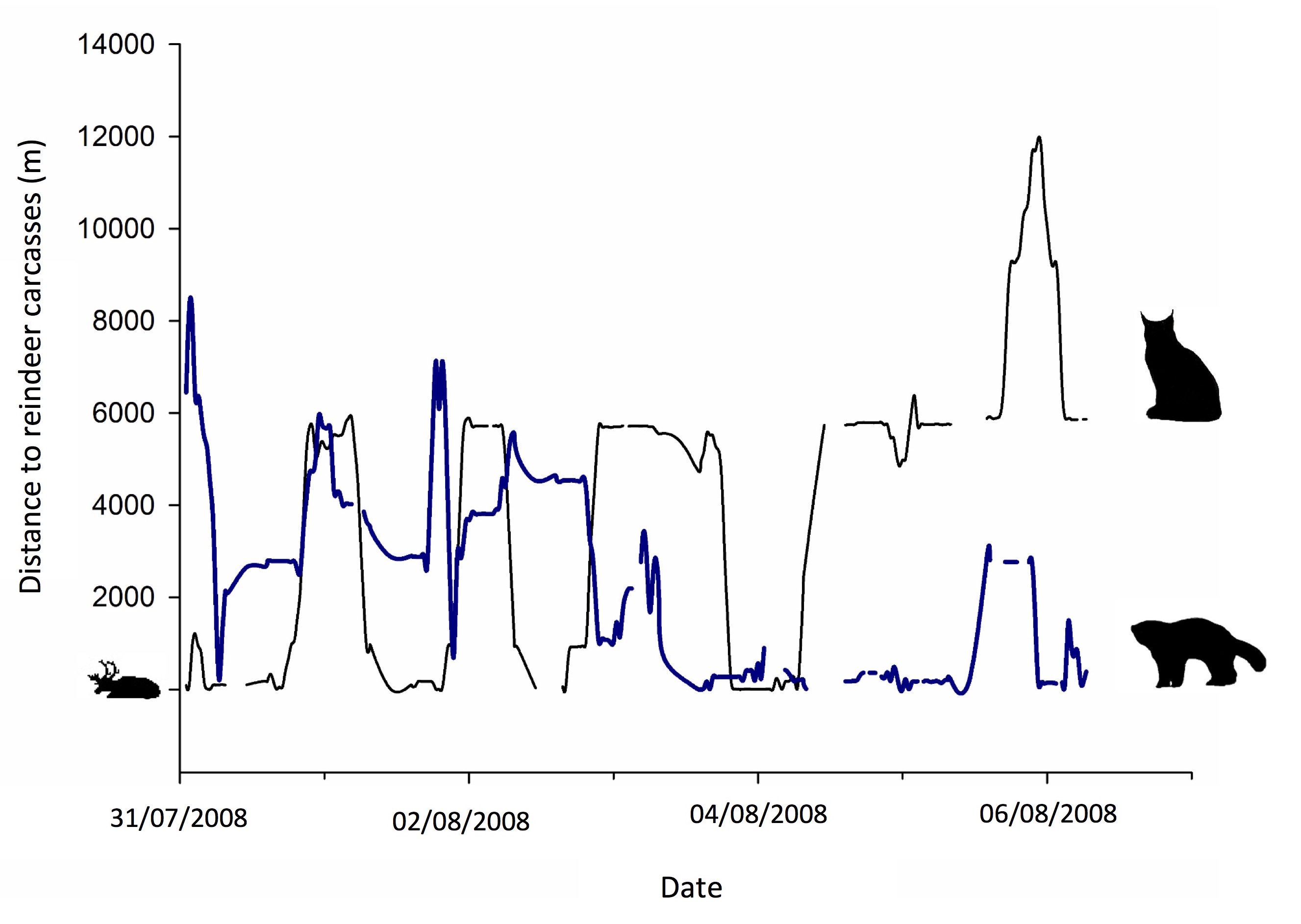


**Figure S2.** Effect of the time (h) elapsed between the estimated date and time of a lynx kill event and the date and time when the animal location was taken (namely exposure time) on the locations of lynx and wolverines in relation to the location of reindeer carcasses. Shaded area represents 95% confidence intervals. See also Table S1.

**
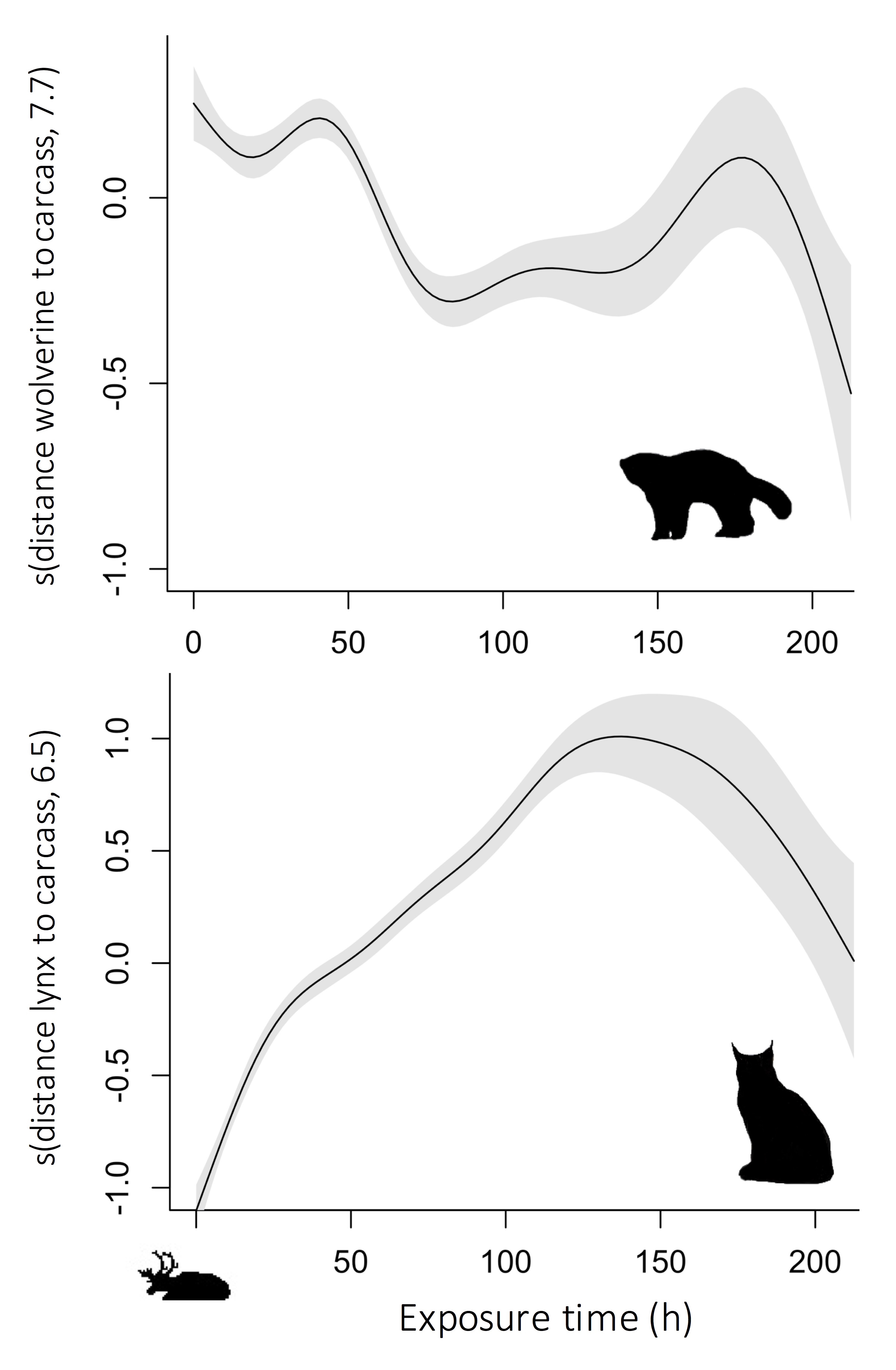
**

**Figure S3.** Histogram showing the distribution of distances between lynx locations and the reindeer carcasses (n total days sampled = 61, all carcasses pooled). Grey bars denotes cases <1,000 m

**
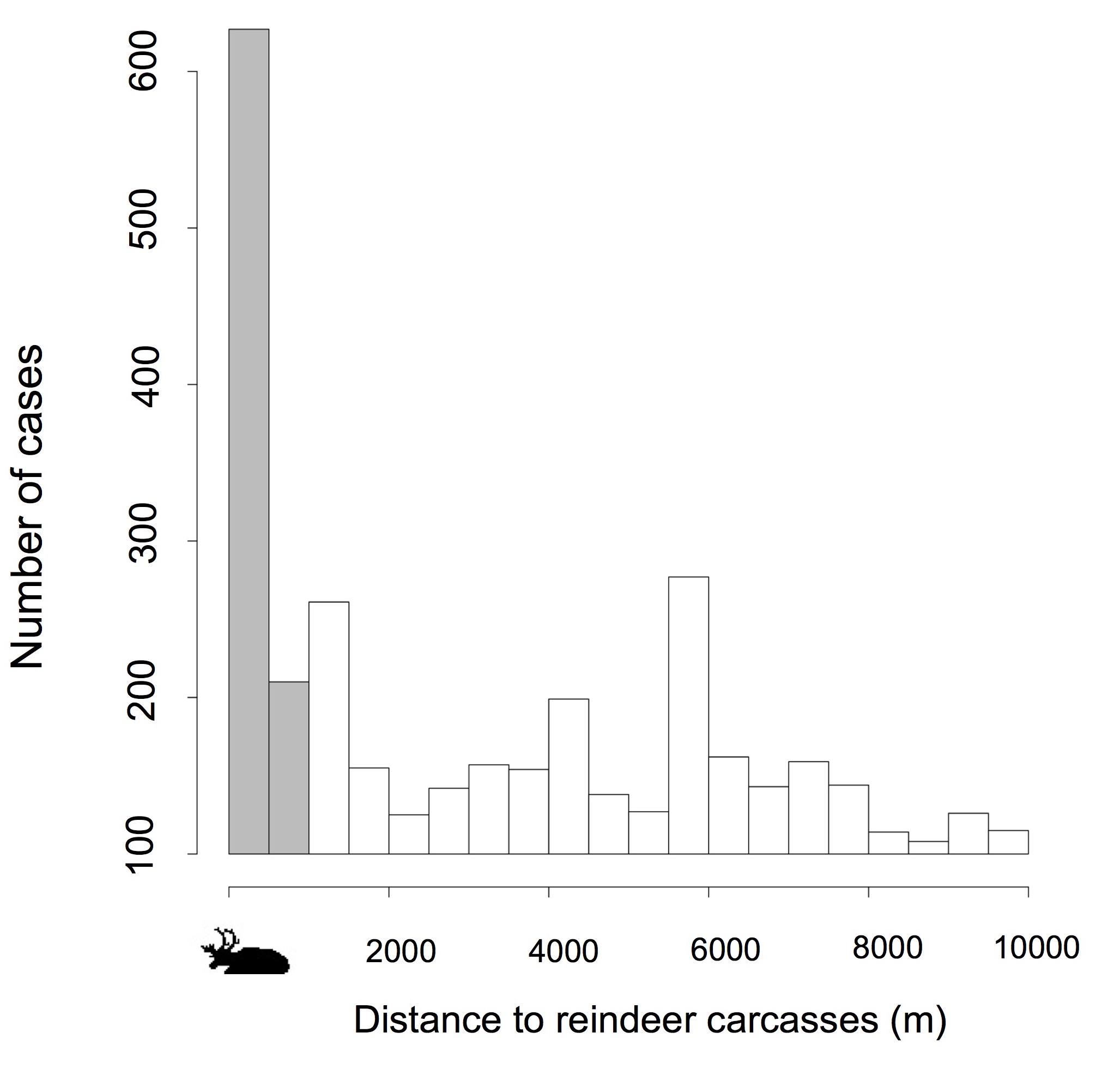
**

**Figure S4.** Histogram showing the distribution of distances between wolverine locations and the reindeer carcasses (n total days sampled = 61, all carcasses pooled). Grey bars denotes cases <1,000 m

**
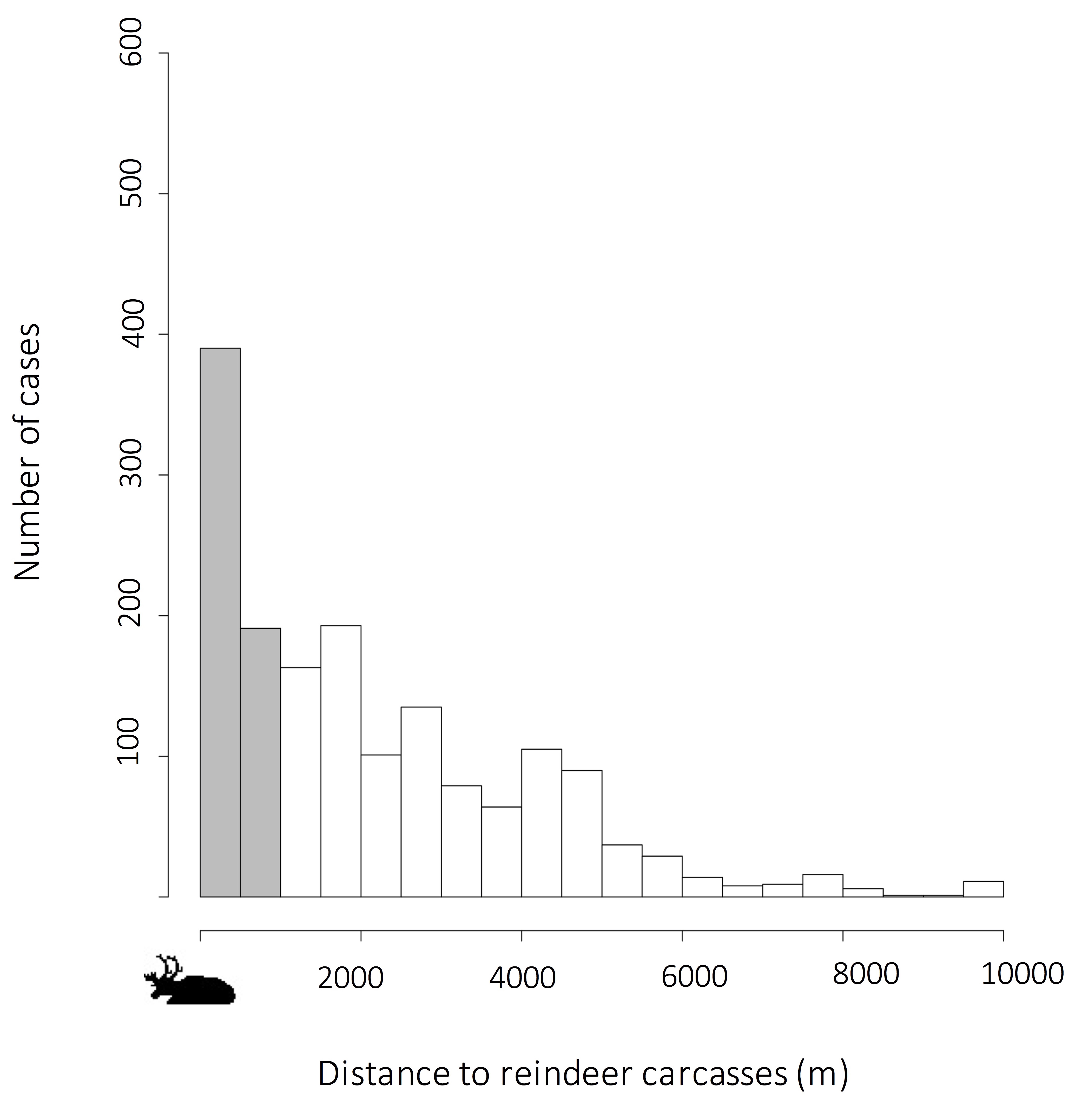
**

**Figure S5.** Histogram showing the distribution of distances between simultaneous locations of lynx and wolverines (n total days sampled = 61, all carcasses pooled). Grey bars denotes cases <1,000 m

**
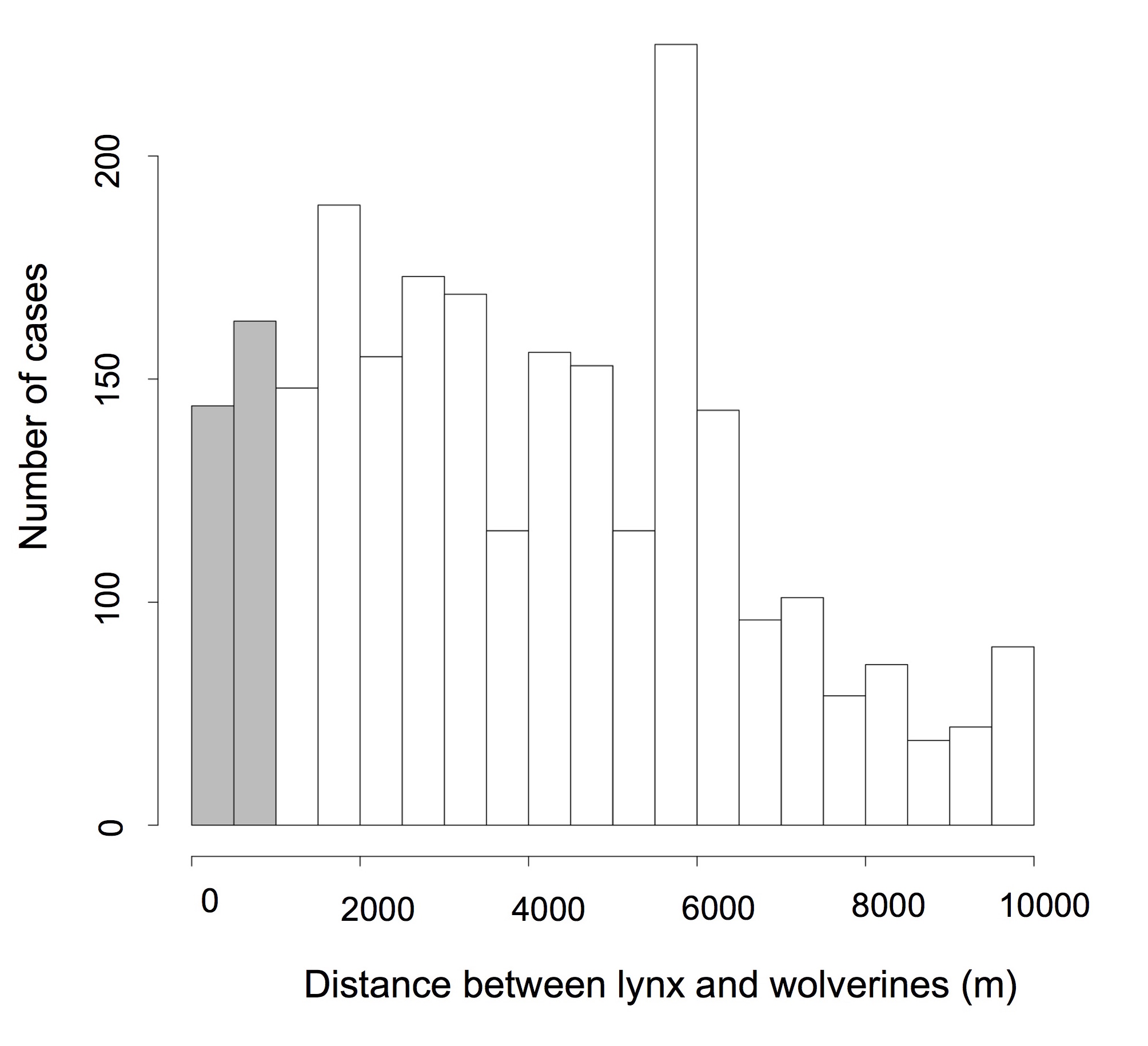
**

**Table S1.** Detailed results of generalized additive mixed models that test for non-linear relationships between wolverine and lynx locations regarding to the location of reindeer carcasses and the time (h) elapsed between the estimated date and time of a lynx kill event and the date and time when the animal location was taken (namely exposure time). Since a non-linear relationship between exposure time and the position of predators was expected, and we use multiple locations from the same individual we built generalized additive mixed models (GAMMs) with Gaussian error distribution and identity link. The exposure time was modeled as smoothed parameter. The appropriate degrees of freedom of the smoothers were selected automatically using cross-validation (Wood 2006). GAMMs were fitted using the ‘*gamm4*’ package (Wood & Scheipl 2013) within the R 3.1 statistical software (R Core Team 2014). The identity of the dyad and the carcasses were treated as random factors in both models. To run these models we only used wolverine and lynx locations less than 10,000 m from the reindeer carcasses (1,643 simultaneous locations).

|  | WOLVERINE | | | | LYNX | | | |
| --- | --- | --- | --- | --- | --- | --- | --- | --- |
|  | estimate | se | edf | *p* | estimate | se | edf | *p* |
|  |  |  |  |  |  |  |  |  |
| *Parametric coefficients:* |  |  |  |  |  |  |  |  |
| Intercept | 3.06 | 0.05 |  | <0.001 | 3.08 | 0.15 |  | <0.001 |
| *Smooth terms:* |  |  |  |  |  |  |  |  |
| Exposure time (h) |  |  | 7.75 | <0.001 |  |  | 6.45 | <0.001 |
| R^2^ (adjusted) | 0.07 |  |  |  | 0.25 |  |  |  |

*edf = estimated degrees of freedom.*

**References**

R Core Team. R: A language and environment for statistical computing. R Foundation for Statistical Computing, Vienna, Austria. URL <http://www.R-project.org/>. (2014).

Wood, S.N. *Generalized Additive Models: An Introduction with R*. (Chapman and Hall/CRC Press, 2006).

Wood, S. & Scheipl, F. gamm4: Generalized additive mixed models using mgcv and lme4. R package version 0.2-2. <http://CRAN.R-project.org/package=gamm4>. (2013)

**Table S2.** Best-fitting probability density functions to wolverine reaction distances to lynx encounters.

| Function | Parameter 1 (± se) | Parameter 2 (± se) | AIC | ∆ AIC |
| --- | --- | --- | --- | --- |
| Gamma | scale = 190.78 (52.9) | shape = 2.0 (0.49) | 400.13 | 0 |
| Log-normal | mean = 5.67 (0.14) | sd = 0.79 (0.10) | 402.06 | 1.93 |
| Exponential | rate = 0.0026 (0.0003) | - | 404.94 | 4.81 |
| Normal | mean = 382.7 (48.2) | sd = 259.8 (34.1) | 408.78 | 8.65 |
| Student *t* | df = 1.25 (0.28) | ncp = 120.9 (18.3) | 413.46 | 13.33 |
